# Imaging and Spectroscopy of Natural Fluorophores in Pine Needles

**DOI:** 10.3390/plants7010010

**Published:** 2018-02-02

**Authors:** Lloyd Donaldson, Nari Williams

**Affiliations:** 1 Biotransformation, Scion, Private Bag 3020, Rotorua 3010, New Zealand; 2 Forest Protection, Scion, Private Bag 3020, Rotorua 3010, New Zealand; nari.williams@scionresearch.com

**Keywords:** autofluorescence, lignin, suberin, ferulate, flavonoid, terpene, chlorophyll, pine needle, spectroscopy

## Abstract

Many plant tissues fluoresce due to the natural fluorophores present in cell walls or within the cell protoplast or lumen. While lignin and chlorophyll are well-known fluorophores, other components are less well characterized. Confocal fluorescence microscopy of fresh or fixed vibratome-cut sections of radiata pine needles revealed the presence of suberin, lignin, ferulate, and flavonoids associated with cell walls as well as several different extractive components and chlorophyll within tissues. Comparison of needles in different physiological states demonstrated the loss of chlorophyll in both chlorotic and necrotic needles. Necrotic needles showed a dramatic change in the fluorescence of extractives within mesophyll cells from ultraviolet (UV) excited weak blue fluorescence to blue excited strong green fluorescence associated with tissue browning. Comparisons were made among fluorophores in terms of optimal excitation, relative brightness compared to lignin, and the effect of pH of mounting medium. Fluorophores in cell walls and extractives in lumens were associated with blue or green emission, compared to the red emission of chlorophyll. Autofluorescence is, therefore, a useful method for comparing the histology of healthy and diseased needles without the need for multiple staining techniques, potentially aiding visual screening of host resistance and disease progression in needle tissue.

## 1. Introduction

Plant tissues contain many fluorescent compounds [1]. This natural fluorescence is called autofluorescence and can be used for imaging cells and tissues. Where fluorophores exist in spatial isolation, it is possible to identify the fluorophore by histochemistry, by comparison with purified standards, or potentially by using other forms of spectroscopy such as FTIR or Raman microspectroscopy [1,2,3,4]. Autofluorescence can be used to image tissues even when the fluorophore has not been identified with the advantage of high contrast and spatial resolution [4,5]. In some cases, autofluorescence may either complement or interfere with staining or labeling protocols, especially when the fluorescence is relatively bright as is the case with chlorophyll [6,7]. As more plant fluorophores are characterized, autofluorescence will become an increasingly important imaging tool in plant science.

Fluorescence has the advantage of high spatial resolution for imaging applications, but fluorescence spectra have limited direct linkage to chemical characteristics. Fluorescence spectra have a fixed emission peak and intensity changes as the excitation wavelength is varied in relation to the absorption spectrum of the fluorophore [8]. In terms of using autofluorescence as a histological tool for the microscopic characterization of tissues, the most practical approach is to use sequential excitation with a range of wavelengths over the ultraviolet (UV) and visible light spectrum. Since most modern fluorescence microscopy is performed with confocal microscopes and laser-based illumination, the optimum excitation wavelength and emission range need to be determined for a particular fluorophore in relation to the available range of laser emission lines. Although lasers with continuously variable emission are now available, there are few studies where the excitation/emission characteristics of a plant fluorophore have been fully characterized in situ [9].

Autofluorescence can be characterized using three parameters: emission spectrum, intensity (quantum yield), and fluorescence lifetime [8,10]. Fluorescence lifetime spectroscopy is potentially very useful in plant histology but, as of yet, its use has been rather limited [11,12]. There are two main examples of fluorescence spectroscopy being used in plant science involving studies of chlorophyll or lignin. Chlorophyll fluorescence can readily be measured using handheld devices on living leaves and this measurement is routinely used in physiological studies [13]. Lignin fluorescence has been extensively studied because of its importance in wood science. In microscopic applications, lignin autofluorescence has been used in studies of reaction wood formation [14,15], cell wall porosity [16], and plant biomass deconstruction [17]. Lignin is characteristic of xylem tissue occurring as a complex polymer containing a variety of fluorophores [18]. Its fluorescence is generally considered to be the combined emission of individual chemical structures, as these never occur in spatial isolation at the micro- or nanoscales. There are two types of fluorophore associated with lignin: phenolic structures that are excited by UV light and have blue emission, and conjugated structures excited by visible light with broad emission across the visible light spectrum into the red and far-red wavelengths [14]. Lignin fluorescence is characterized by its broad range of excitation and emission wavelengths, and while relatively weak, it is certainly sufficient for imaging of cell walls in wood and other tissues [15].

Discrimination of fluorophores in plant tissues can be achieved in two ways. First, by sequentially imaging tissue with multiple excitation wavelengths, different fluorophores may exhibit emission wavelengths with different relative intensities and hence may appear as different colors in fluorescence images. In other cases, fluorophores may have strongly overlapping emissions that require spectral imaging and linear unmixing to achieve separation [19]. Some fluorophores have a pH-dependent emission that can provide discrimination [19,20].

In the present study, we examine the autofluorescence of healthy pine needles compared to chlorotic and necrotic needles. We provide a characterization of cell wall components including lignin, suberin, ferulate, and flavonoids, extractives such as oleoresin and terpenes, and chlorophyll, including a comparison between histochemistry and autofluorescence where possible.

## 2. Results

### 2.1. Histochemistry

**Figure 1:**
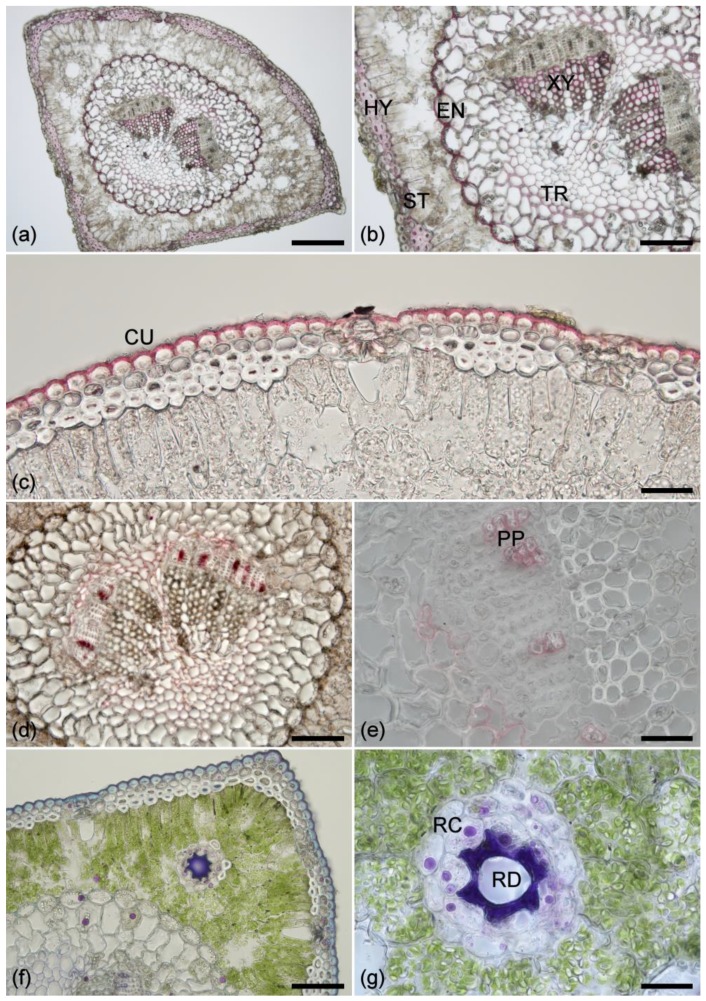
(**a**) Phloroglucinol staining of a cross section from a healthy needle showing lignified tissue in pink and red. Scale bar = 0.2 mm. (**b**) An enlarged view showing phloroglucinol staining of xylem (XY), endodermis (ED), stomata (ST), hypodermis (HY), and transfusion tissue (TR). Scale bar = 100 µm. (**c**) Sudan IV staining of the cuticle (CU). Scale bar = 50 µm. (**d**) Sudan IV staining showing lipid-containing cell walls (suberin) located in the phloem parenchyma and transfusion tissue. Scale bar = 100 µm. (**e**) Higher magnification showing the phloem parenchyma (PP) cell walls stained with Sudan IV. Scale bar = 50 µm. (**f**) NADI staining showing isoprenoids (purple) in resin canal parenchyma, endodermis, transfusion tissue, and rarely in the mesophyll; lipids (blue) in cuticle and epidermis, transfusion and phloem parenchyma, and in the contents of some hypodermal cells; and oleoresin (violet) within the resin duct. Scale bar = 100 µm. (**g**) Higher magnification view of isoprenoids (purple) in resin canal parenchyma (RC) and oleoresin (violet) within the resin duct (RD). Scale bar = 50 µm.

Phloroglucinol staining of healthy, mature *Pinus radiata* needles indicates that xylem, endodermis, and guard cells are strongly lignified (Figure 1a,b). The hypodermis has a moderately lignified primary cell wall but the inner secondary cell wall is unlignified or weakly lignified. The epidermis and transfusion tissue are weakly lignified. Sudan IV staining of fresh healthy needles indicates the presence of lipid-based cell wall components (cutin and suberin) in the phloem parenchyma, transfusion tissue, and cuticle (Figure 1c–e). NADI reagent indicates the presence of isoprenoids (purple) in the endodermis, transfusion tissue, and in resin canal parenchyma in fresh, healthy needles (Figure 1f,g). Lipids are stained violet-blue in oleoresin within resin ducts, and in cell walls within the cuticle, epidermis, transfusion parenchyma, and phloem parenchyma.

The vanillin/HCl test for condensed tannins was negative in fresh or fixed healthy needles and in necrotic needles. The nitrosylation test for catechols gave a strong reaction with mesophyll contents in fresh, healthy needles, but rather than the expected red color, the reaction gave a brown/black color and so must be considered inconclusive.

### 2.2. Lignin Fluorescence

**Figure 2:**
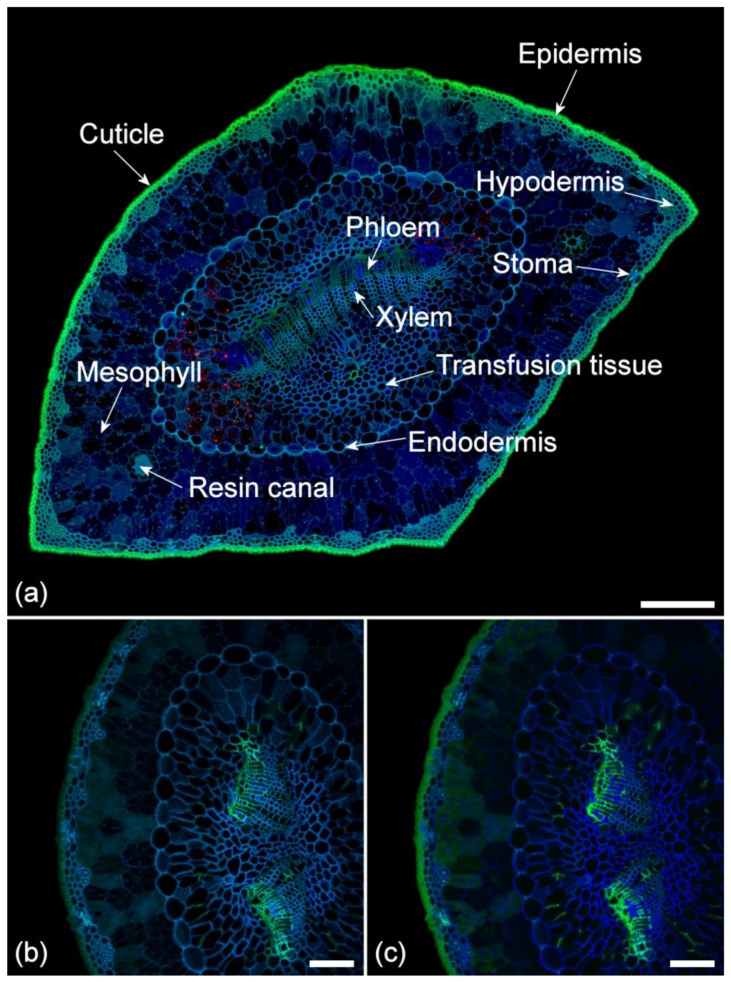
(**a**) Anatomy of a chlorotic pine needle showing autofluorescent structures. The red fluorophore is residual chlorophyll in the transfusion tissue. Sequential excitation at 355, 488, and 633 nm with corresponding emission at 400–500 (blue), 500–570 (green), and 650–750 nm (red). Scale bar = 200 µm. (**b**) Autofluorescence of a healthy needle using spectral imaging to more precisely detect the green ferulate fluorescence in the phloem tissue and to exclude chlorophyll fluorescence. Excitation 355 nm with emission at 420–460 nm (blue) and 495–550 nm (green). Scale bar = 100 µm. (**c**) Autofluorescence of a healthy needle using blind spectral unmixing to more precisely detect the green ferulate fluorescence in the phloem and to exclude chlorophyll fluorescence. Excitation 355 nm with emission at 400–800 nm. Scale bar = 100 µm.

Lignin is a phenylpropanoid and, in pine, consists of a polymer of coniferyl and p-coumaryl alcohols [21]. In pine needles, lignin is most easily identified as the dominant fluorophore in xylem (Figure 2). Needles contain a number of UV excited blue fluorophores (Table 1), so identifying the lignin in tissues outside of the xylem is complex. Because lignin has a wide excitation range, sequential excitation with UV and blue light results in emission in the blue and green range which separates lignin from the other blue fluorophores in needles (Table 1). Lignin is also brighter than other blue fluorophores, at least in tissues where it is abundant (Figure 2). In addition to xylem, lignin fluorescence can be seen in the endodermis that surrounds the vascular tissue. Guard cells in the stomata also show strong lignification in an area of highly thickened cell wall. Lignin fluorescence can also be detected in the hypodermis, a layer of thick-walled sclerenchymatous cells below the epidermis. In this tissue, the primary cell wall and middle lamella are highly lignified, but the secondary cell wall may be either slightly lignified or may have weak or no lignification varying among individual cells. Some cells within the transfusion tissue surrounding the xylem and phloem including transfusion tracheids are weakly lignified. Observations using autofluorescence agree with those from phloroglucinol staining (Figure 1a,b), but autofluorescence is more sensitive in tissues with weak lignification.

### 2.3. Suberin Fluorescence

Suberin is a polymer of aliphatic and aromatic (hydroxycinnamic acids—ferulate) components, but its exact composition is poorly understood [22]. Suberin fluorescence is similar to lignin with intense blue fluorescence under UV excitation. However, suberin is only weakly fluorescent with blue excitation, and hence with sequential excitation, appears distinctly blue when compared to the blue/green fluorescence of lignin (Figure 2) (Table 1). In pine needles, suberin can be seen in phloem parenchyma cell walls and in bundles of parenchyma cells adjacent to the phloem and scattered throughout the transfusion tissue especially in the region between the two vascular bundles where present (Figure 2). Observations using autofluorescence are more specific than those from Sudan IV or NADI, staining both of which also stain the cuticle in addition to suberized tissues (Figure 1).

### 2.4. Ferulate Fluorescence

Ferulic acid may occur in cell walls where it is usually esterified to polysaccharides, emitting a very weak blue fluorescence with UV excitation at neutral pH that characteristically changes to stronger green emission under conditions of high pH such as in the presence of ammonia [20] (Table 1). In pine needles, ferulate can be detected in phloem cell walls especially in the primary wall/middle lamella with very weak emission in the secondary wall (Figure 2b,c), varying significantly among individual needles from quite strong to relatively weak in intensity. In some needles, it was not detected. Sporadic deposits of ferulate can sometimes be observed within the transfusion tissue. There are no histochemical tests for ferulate, so its identification in tissue is based solely on demonstrating a pH-dependent change from blue to green fluorescence.

### 2.5. Cuticle Fluorescence

**Figure 3:**
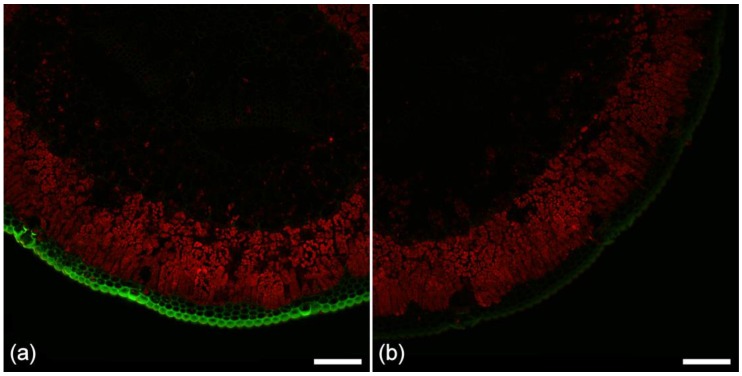
A healthy pine needle showing flavonoid distribution. (**a**) Treated with Naturstoffreagenz A (NA) for 5 min showing localization of flavonoids (green) in the epidermis and hypodermis; (**b**) Untreated control at the same gain setting. The red fluorophore is chlorophyll. Sequential excitation at 488 and 561 nm with corresponding emission at 500–570 (green), and 570–700 nm (red). Scale bar = 100 µm.

The composition of the cuticle is complex with cutin, a waxy polymer of several different fatty acids linked by ester bonds, being a major component. Cutin forms part of the cuticle that covers the external surface of the epidermis in plants [23,24] but is not known to be autofluorescent. Flavonoids are hydroxylated polyphenolic compounds and, in pine needles, are represented by diacylated flavonol glucosides [25]. Flavonoids are autofluorescent and can readily be distinguished from other phenolic fluorophores by treatment with Naturstoffreagenz A (NA), which results in significant brightening of the flavonoid autofluorescence compared to other fluorophores [25]. As shown in Figure 3, flavonoids are localized to the cuticle, epidermal cell walls, and cell contents, and possibly to the hypodermal cell walls. Fluorescence of the cuticle is therefore likely to be due mainly to flavonoids. Hydroxycinnamic acids may also be present in the cuticle but these are weak blue fluorophores that would likely be masked by the stronger green fluorescence from the flavonoids [25]. In pine needles, the cuticle fluoresces with blue excitation, resulting in a typically green emission (Figure 2) (Table 1). Autofluorescence distinguishes fluorescence of the cuticle from suberin, compared to Sudan IV staining, which reacts with both components albeit more intensely with the cuticle (Figure 1c). NADI staining also reacts with the lipid component of the cuticle and epidermis (Figure 1f). The intensity of cuticle fluorescence often varies between the abaxial and adaxial surfaces of the needle being more intense on the outer abaxial (curved) surface (Figure 2).

### 2.6. Other Cell Wall Fluorophores

In addition to the above fluorophores, the primary cell walls of mesophyll cells show weak blue fluorescence. The exact nature of this fluorophore remains undetermined but may represent cell wall-bound phenolic components such as hydroxycinnamic acids (*p*-coumaric acid) [26], flavans, and non-acylated flavonol glucosides [25]. This fluorophore is not responsive to increased pH, thus is distinctive from the ferulate in phloem cell walls.

### 2.7. Extractive Fluorescence

Pine needles contain several types of extractives, including terpenes, isoprenoids, flavonoids, and oleoresin [27,28]. Terpenes in pine are dominated by pinene [27]. In pine needles, extractives are found within the hypodermis, mesophyll, resin canal parenchyma and resin ducts, endodermis, and transfusion parenchyma (Figure 2). There are two types of autofluorescence associated with extractives, deep blue fluorescence located within mesophyll cells and transfusion parenchyma, and brighter blue/green fluorescence located in resin canals/ducts, hypodermis, endodermis, and as small droplets in mesophyll cells.

Isoprenoids are non-fluorescent and occur as distinct droplets identified by NADI reagent, whereas oleoresin in resin ducts fluoresces blue/green with UV/blue excitation. NADI reagent confirms the presence of isoprenoids in resin canal parenchyma, endodermis, hypodermis, and rarely in the mesophyll (Figure 1f).

Mesophyll cells are filled with abundant extractive materials that do not react with NADI reagent confirming the absence of isoprenoids and oleoresin in these cells. Extractives located in the mesophyll, filling the cell lumens fluoresce dark blue (Figure 2). Comparing this component with purified pinene using fluorescence confirmed that both show weak dark blue fluorescence with UV excitation, but this is inconclusive, as many phenolic compounds show generic blue fluorescence under UV excitation. The presence of small droplets of a brighter blue/green fluorophore within mesophyll cells suggests the presence of a mixture of extractives in these cells. Flavonoid extractives are localized to the epidermal cell lumens using NA (Figure 3).

### 2.8. Chlorophyll Fluorescence

**Figure 4:**
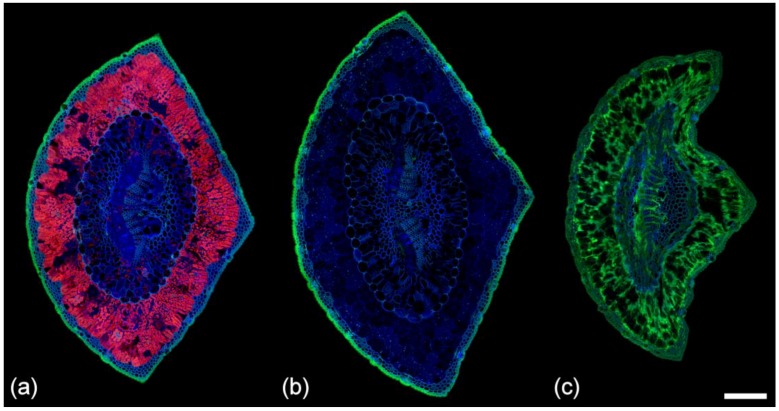
Autofluorescence in (**a**) healthy; (**b**) chlorotic; and (**c**) necrotic needles from *Pinus radiata*. Sequential excitation at 355, 488, and 633 nm with corresponding emission at 400–500 (blue), 500–570 (green), and 650–750 nm (red). Note the absence of resin canals in these three needles from the same tree. Fluorescence associated with ferulate is also absent from the phloem in these needles. Both features were found to be variable. Scale bar = 200 μm.

Chlorophyll is a porphyrin with very strong red fluorescence [29]. Chlorophyll is excited by all laser excitation wavelengths from 355 to 633 nm (Table 1); we used 633 nm excitation in this study. Chlorophyll has two distinct emission peaks dominated by photosystem II at 685 nm with a shoulder from 700 to 750 nm. In pine needles, chlorophyll occurs within chloroplasts mainly in the mesophyll, but smaller amounts are also found in the transfusion and vascular tissues (Figure 2 and Figure 4). Chlorophyll fluorescence is the only red fluorophore in pine needles but is so intense relative to blue and green fluorophores that it can obscure other components, particularly in the mesophyll. Chlorophyll is readily extracted with ethanol but this treatment will also remove or modify extractives and potentially affect some cell wall fluorophores. However, because chlorophyll fluorescence emission is at longer wavelengths compared to other fluorophores, it can easily be excluded from images by blocking the red emission channel above 640 nm or by spectral unmixing. Chlorophyll fluorescence is greatly reduced in chlorotic needles and absent in necrotic needles (Figure 4). Imaging of chlorotic needles confirmed that there are no other red fluorophores in pine needles when chlorophyll fluorescence was minimized. No other pigments (carotene, xanthophyll) were distinguishable.

### 2.9. Comparison of Spectra

**Figure 5:**
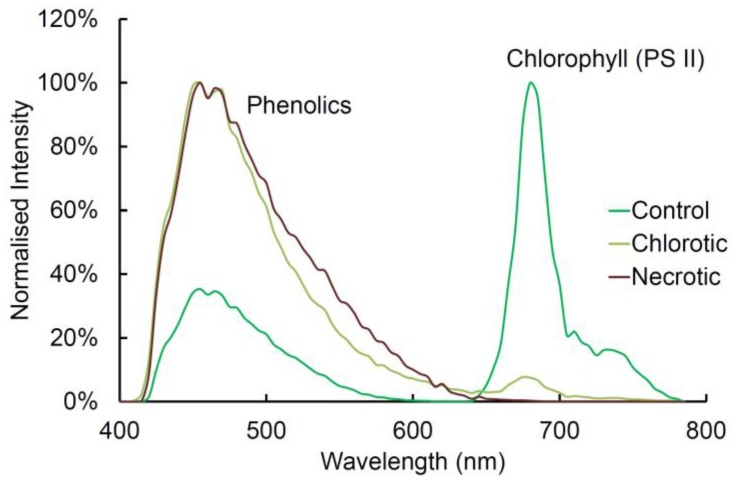
Fluorescence spectra of normal, chlorotic, and necrotic needles from radiata pine. Excitation at 355 nm with emission at 400–800 nm. There are two groups of fluorophores present, blue-green emitters (suberin, lignin, ferulates, flavonoids, and extractives) and orange/red emitters (chlorophyll, photosystem II (PSII)).

Sequential excitation can be used for imaging autofluorescence as described above. As an alternative, spectral imaging with a single excitation wavelength can be used, either to compare the spectra of different fluorophores with the same excitation wavelength, or to create images of specific fluorophores using spectral unmixing (Figure 2b,c). Spectra from healthy, chlorotic, and necrotic needles show significant differences related to loss of chlorophyll and to the increase in green fluorescence from necrotic mesophyll cells associated with the browning of needles (Figure 4 and Figure 5). Chlorosis involves gradual loss of chlorophyll with no obvious changes in other fluorophores. In necrotic needles, there is a progressive loss of chlorophyll together with the formation of a novel blue excited green fluorophore in the mesophyll. Necrotic needles may show other changes such as accumulation of extractives in the endodermis and transfusion tissue, as well as shrinkage and collapse of tissue due to desiccation.

**Figure 6:**
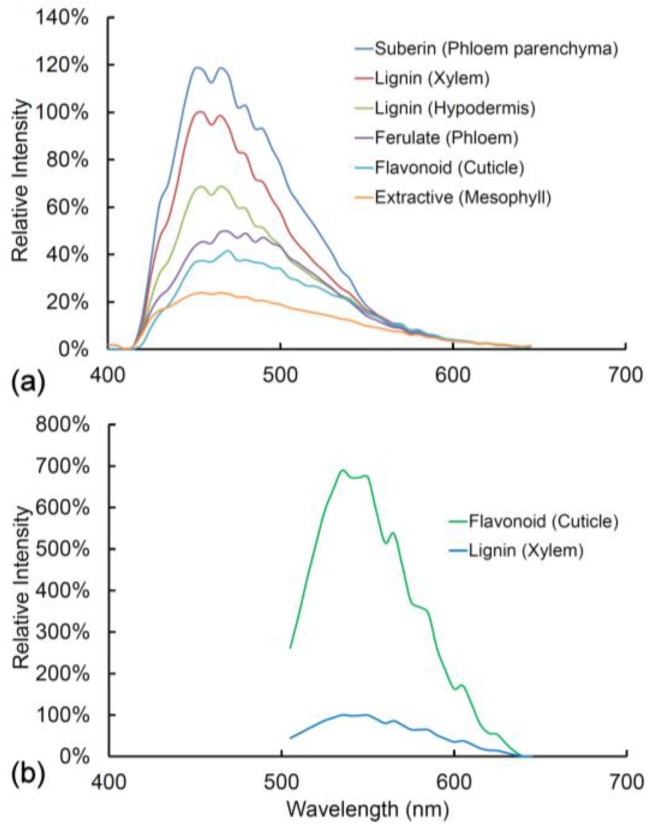
(**a**) Fluorescence spectra from a fresh healthy pine needle with 355 nm excitation showing relative intensities among fluorophores/tissues compared to lignin located in the xylem; (**b**) Fluorescence spectra from lignin (xylem) and flavonoids (cuticle) in a fresh healthy pine needle with 488 nm excitation. The two spectra are identical in shape, but flavonoids have a much higher intensity than lignin and hence appear green rather than blue in sequentially excited fluorescence images.

**Figure 7:**
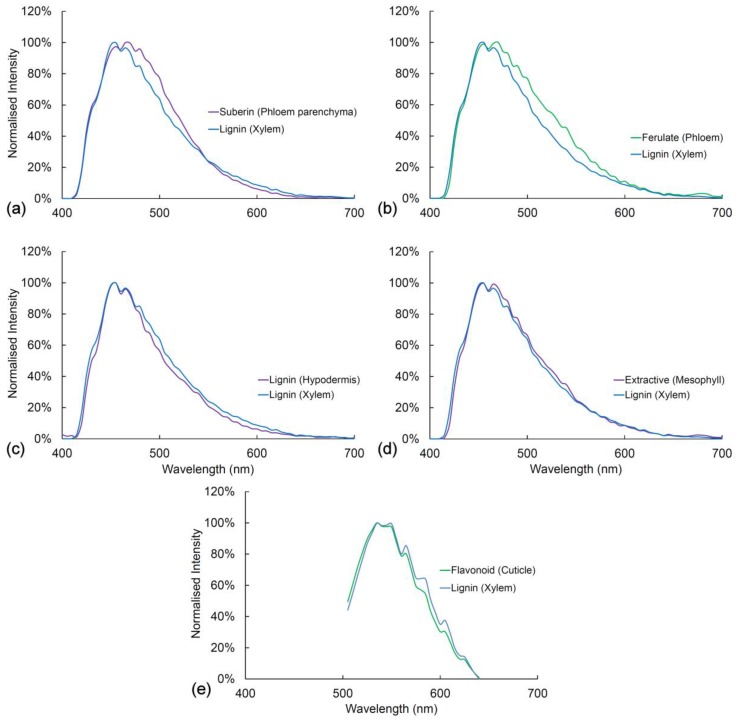
Comparison between lignin and other cell wall fluorophores. (**a**) Lignin and suberin have similar spectra with UV excitation (355 nm), $\chi^{2}=31.5\;ns$; (**b**) Lignin and ferulate have significantly different spectra with UV excitation, $\chi^{2}=59.1***$; (**c**) Lignin from xylem and hypodermis have identical spectra with UV excitation, $\chi^{2}=32.4\;ns$; (**d**) Lignin from xylem and extractives in the mesophyll have identical spectra with UV excitation, $\chi^{2}=15.4\;ns$; (**e**) Lignin and flavonoids have identical spectra with blue excitation (488 nm), $\chi^{2}=11.9\;ns$. *ns* = non-significant, * = *p* < 0.05, ** *p* < 0.01, *** *p* < 0.001.

In Figure 6 and Figure 7, we compare the spectra of blue and green fluorophores with lignin as a reference spectrum. This allows an assessment of relative intensity (Figure 6). Alternatively, spectra can be compared after intensity differences are removed by normalization to detect spectral differences (Figure 7). Relative intensity may relate to the amount of fluorophore or it may relate to differences in quantum yield between fluorophores. The differences in intensity between different lignified tissues are strongly related to the amount of lignification with more highly lignified xylem being brighter than weakly lignified hypodermis for example.

Fluorophores can be ranked according to intensity (Figure 6). With UV excitation, suberin is the brightest fluorophore followed by lignin in the xylem, lignin in the hypodermis, ferulate in the phloem, flavonoids in the cuticle, and extractives in the mesophyll. Ferulate in the phloem (excitation 355 nm) and flavonoids in the cuticle (excitation 488 nm) have very low blue emission and strong green emission, thus accounting for their bright green color in the sequential excitation images (Figure 2). After normalization of intensity differences, the spectra of all the blue/green fluorophores are very similar to that of lignin (Figure 7). Ferulate shows a significant difference ($\chi^{2}=59.1***$) from lignin with a shift towards green fluorescence under UV excitation. With blue excitation, flavonoids are seven-fold brighter than lignin (Figure 6b) even though the emission spectrum is not significantly different ($\chi^{2}=11.9\;ns$) (Figure 7e).

### 2.10. Fresh vs. Fixed Tissue

**Figure 8:**
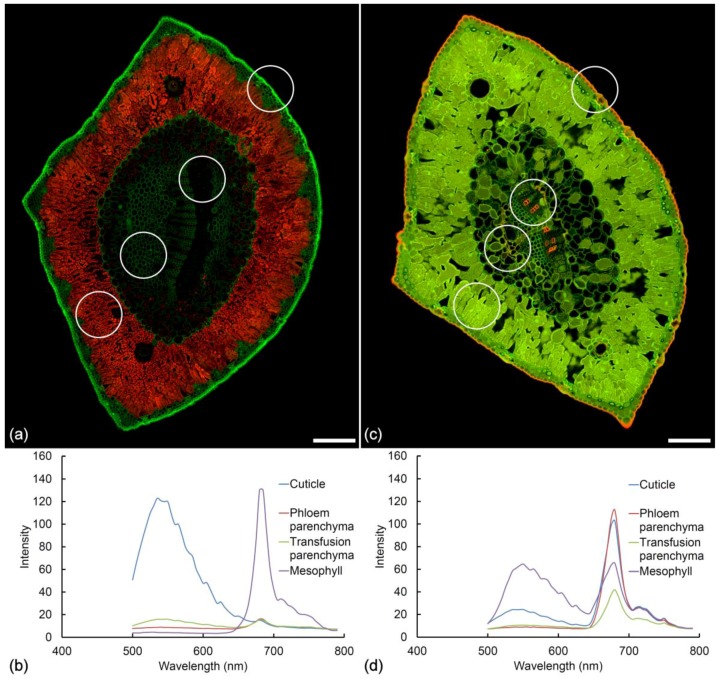
Autofluorescence of a healthy needle before and after fixation in Formalin Aceto Alcohol (FAA). Sequential excitation at 488 and 561 nm with corresponding emission at 500–570 and 570–700 nm. (**a**) Fresh pine needle and (**b**) emission spectra from the cuticle, phloem parenchyma, transfusion parenchyma, and mesophyll, the later showing intense chlorophyll fluorescence. Scale bar = 200 µm. (**c**) Pine needle fixed in FAA for 1 month. The cuticle, phloem parenchyma, and transfusion parenchyma exhibit a new fluorophore showing orange emission while extractives in the mesophyll now show strong green fluorescence. Scale bar = 200 µm. (**d**) Spectra acquired from the cuticle, phloem parenchyma, and transfusion parenchyma with 488 nm excitation indicate the orange fluorophore is chlorophyll with its characteristic twin peaks, redistributed by the ethanol-based fixative.

Fixation with FAA (Formalin Aceto Alcohol) may result in modification of needle autofluorescence (Figure 8). Two distinct changes are noted in healthy pine needles after fixation in FAA. Firstly, the extractives inside the mesophyll cells become much more fluorescent and the emission moves from blue to green/yellow. Secondly, the cuticle changes its fluorescence from green to orange. Treatment with FAA fixative results in removal and redeposition of chlorophyll in lipid-containing tissues including the cuticle, transfusion tissue, and phloem parenchyma (Figure 8c). This chlorophyll “staining” is similar to Sudan IV staining but is much more intense. Chlorophyll redistribution results in characteristic chlorophyll fluorescence spectra detectable throughout the needle tissue to varying degrees post-fixation (Figure 8d). Treatment with ethanol alone produces the same changes.

## 3. Discussion

Our study was aimed at using autofluorescence to characterize needle histology as a potential screening tool for possible resistance to red needle cast disease in radiata pine caused by *Phytophthora pluvialis* [30]. The advantage of using autofluorescence is to avoid the need for multiple staining protocols resulting in a single image that can be used to evaluate differences among tree genotypes or to study disease progression. Using sequential UV, blue and red excitation, we were able to image at least six distinct fluorophores in fresh vibratome sections of needles. Further information was obtained using fixed needles where some fluorophores changed their emission spectrum or became redistributed (Figure 8). The disadvantage of imaging fresh needles is the need to complete imaging within a few days of collection.

Relatively few studies have characterized plant autofluorescence [1,2,3,6,19,20,31,32,33,34]. Lignin is the only cell wall-associated fluorophore that has been investigated in detail [19], although some limited information is available for hydroxycinnamic acids such as coumarate and ferulate [20,26], as well as suberin [33].

In pine needles, xylem and endodermis are highly lignified and show fluorescence identical to lignin in the secondary xylem of the stem [19]. Other tissues are less well lignified, the hypodermis has quite variable lignification, while the epidermis and transfer tissue are weakly lignified. Compared to phloroglucinol staining, autofluorescence gives comparable results but seems more sensitive to low lignin levels in the transfusion tissue, for example. Lignin is separated from other cell wall fluorophores by its characteristically wide emission range. The fluorescence spectrum of lignin has a λmax of 455 nm with UV excitation and 535 nm with blue excitation corresponding to emission from phenolic and conjugated structures respectively [14]. Lignification of the xylem, endodermis, and hypodermis showed no obvious variation among different needles. Other studies have found differences in degree of lignification of the endodermis between different species using both lignin autofluorescence and phloroglucinol staining [34].

Transfusion tissue contains a number of cell types including transfusion tracheids and parenchyma cells with variable cell wall composition. The transfusion tissue acts as a pathway for water movement from the xylem into the mesophyll spaces and the outside environment via the stomata. Despite this important physiological function, relatively little is known about its composition. Autofluorescence demonstrates that transfusion tracheids are very weakly lignified compared to xylem tracheids. Some transfusion parenchyma cell walls contain suberin, which presumably acts to restrict water loss from the xylem into the surrounding needle tissue. Some cell walls within this tissue may contain both lignin and suberin, although this is difficult to determine by autofluorescence alone.

The highly lignified endodermis may also restrict water movement into the mesophyll, forming a Casparian strip, but it also represents a lignified barrier to invasion of the vascular tissue by pathogens [35,36]. We did not find evidence for suberin in the endodermis of radiata pine [4]. Interestingly, the resin canal epithelium in needle resin canals appears to lack the suberization found in resin canals in secondary xylem [33]. Suberin is much easier to detect by autofluorescence than by Sudan IV staining, which tends to be weak in conifer tissues, especially in comparison to cutin, which reacts strongly with this stain (Figure 1c).

Ferulates have been detected esterified to primary cell walls in all families of gymnosperms [37]. In pine needles, cell wall bound ferulates are detected in phloem cell walls. This fluorophore varies considerably among needles from strong to very weak emission and may even be absent in some needles, although the reasons for this variability are not immediately apparent. Ferulate esters, like other phenylpropanoids, can be regulated as part of stress responses, so the variability we observed may reflect the stress states of needles [38].

Flavonoid fluorescence did not show obvious variability among needles, but there was a prominent variation between the abaxial (curved) and adaxial surfaces of the needle with significantly less flavonoid fluorescence on the adaxial surfaces in most needles examined. This might have a significant effect on interactions between pathogens and the needle surface.

The distribution of phenolic compounds in conifer needles has been studied in some detail [2,25]. Phenolic fluorophores may be bound to cell walls [26]. In spruce needles, blue excited autofluorescent flavonoids have been localized in the lumens of needle epidermal cells, where they were described as diacylated flavonol glucosides after treating with NA [2]. In Scots pine, analysis of enzymatically isolated needle epidermis also detected diacylated flavonol glucosides, representing 90% of the total needle content [25]. Flavonoids act as UV absorbers and antioxidants protecting needles from damage due to sunlight exposure [39]. Similar fluorescence was observed in pine needles in the present study with flavonoids localized in the cuticle, epidermal cell walls, and lumens, as well as in hypodermal primary cell walls. It seems that epidermal and hypodermal cell walls have a complex composition with indications of lignin, lipids, and flavonoids being present.

Terpenes and oleoresin are part of the needles defense system, so being able to detect their distribution and amount is of particular interest from a pathology perspective [36]. However, differentiation of extractive components using autofluorescence is limited. Oleoresin in resin ducts is autofluorescent, terpenes such as pinene are very weakly fluorescent while isoprenoids in resin canals, endodermis, and transfusion tissue are non-fluorescent.

Mesophyll cells contain large amounts of faint blue fluorescent material. Based on autofluorescence and histochemistry, there were no clear indications of what this material might be. We were able to exclude oleoresin, lipids, and isoprenoids. Necrotic needles contain dense deposits of green fluorescent material in the mesophyll, which is presumably derived from the blue fluorescent material present in healthy and chlorotic needles. The green fluorophore in necrotic needles has a slightly different fluorescence spectrum from similar deposits in FAA fixed needles and is substantially different from tannin deposits in pine bark (Figure S1). Based on this comparison and the histochemistry, we were not able to determine the exact nature of mesophyll extractives other than to say they are probably polyphenolic. Others have described the formation of tannins in physiologically stressed needles, but we could not confirm that these mesophyll deposits are tannins [39,40,41,42].

The comparative anatomy of pine needles has been studied in detail [43,44], but there have been few comprehensive histological studies of conifer needles. Investigations have focused on pathological or physiological effects such as fungal infection, acid rain, mineral deficiency, or CO_2_ enrichment [2,42,45,46,47]. Autofluorescence in combination with histochemistry has been used to explain differences in the mechanical properties of pine needles between species due to variable lignification of the endodermis [34]. Chlorophyll autofluorescence has been used to detect lesion formation in *Dothistroma* needle blight of radiata pine [47]. We demonstrate the usefulness of autofluorescence in understanding cell wall composition in needle tissues, especially in relation to potential pathological impacts. Autofluorescence, spectral imaging, and fluorescence lifetime imaging [9,10,11,12] will play an increasing role in understanding plant tissue composition.

## 4. Materials and Methods

Young three-year-old *Pinus radiata* D. Don clonal trees growing in pots outdoors at the Scion campus in Rotorua, New Zealand, were examined to determine needle histology comparing healthy needles with chlorotic and necrotic needles resulting from natural senescence. Mature needle fascicles from 10 different trees were collected sequentially and maintained in a fresh condition by immersing the base of the fascicle in water in plastic tubes stored at 4 °C for no more than 5 days before discarding. Fresh needles and needles fixed in FAA (90:5:5 absolute ethanol, glacial acetic acid, formaldehyde) for at least 6 weeks were sectioned transversely at a thickness of 30 μm using a Leica vibratome. Sections were collected from the basal 1 cm of the needle and were stored briefly in water before mounting in 50% glycerol in phosphate buffer at pH9 [19]. Comparisons were also made by mounting in glycerol–buffer at pH 5 or 7. In some cases, sections were treated with or mounted in 0.1 M ammonium hydroxide to enhance localization of cell wall-bound ferulates [20]. To detect fluorescence of flavonoids, fresh sections were mounted in NA reagent [25], and a comparison was made under identical conditions with similar sections in glycerol buffer. Treatment with NA results in a significant brightening of flavonoid fluorescence where present. Sections were examined using a Leica SP5 II confocal fluorescence microscope using sequential excitation at 355, 488, and 633 nm, with respective fluorescence emission at 400–500 (blue), 500–570 (green), and 650–750 (red) nm. In some cases, imaging was performed with 488 and 561 nm excitation, with respective emission at 500–570 and 570–700 nm. A UV corrected 20× immersion objective was used with 80% glycerol in water as the lens immersion medium. Individual images rendered as maximum intensity projections were montaged to provide a high-resolution view of the whole needle cross-section using Microsoft Image Composite Editor software.

For spectral imaging, fresh un-fixed sections were excited at 355 nm and spectral image sequences were acquired from 400–800 nm using a slit width of 10 nm and a scan interval of 5 nm. Some spectra were acquired from fixed sections using 488 nm excitation with emission from 500–800 nm. Regions of interest corresponding to cuticle, hypodermis, mesophyll, phloem, and xylem from spectral image sequences were converted into spectra using Digital Optics V++ software (Auckland, New Zealand) using an image threshold based on the average brightness [48]. This procedure was intended to preserve the fluorophore brightness relative to other tissues by excluding non-fluorescent cell lumens from intensity measurements. Intensity comparisons were made relative to lignin fluorescence in the xylem of the vascular bundle from the same image sequence. Spectral measurements were performed using three replicates which were found to be highly repeatable. Differences between spectra were determined using a $\chi^{2}$ test [49] (*ns* = non-significant, * = *p* < 0.05, ** *p* < 0.01, *** *p* < 0.001). Spectral unmixing was performed using the Poisson NMR application in ImageJ software (Bethesda, MD, USA) [50].

Some fresh needle sections were treated with phloroglucinol to localize lignin. A 2% solution of phloroglucinol in 95% ethanol was freshly prepared and stored in the dark at 4 °C. Immediately prior to use, several drops of concentrated HCl were added to 0.5 mL of phloroglucinol, and sections were treated for 2 min followed by washing in 95% ethanol. Sections were mounted in 95% ethanol for microscopy [51]. Sudan IV was prepared as a saturated solution in 95% ethanol. Sections were stained for 1 h before mounting in glycerol–buffer at pH7 [52]. NADI stain was used to identify isoprenoids and lipids in fresh sections by staining for 1h at room temperature. NADI reagent selectively stains isoprenoids purple and lipids blue. The reagent was prepared by mixing 0.5 mL of a 1% α-naphtol solution in 40% alcohol, 0.5 mL of 1% dimethyl-p-phenylenediamine chloride in water, and 49 mL of phosphate buffer 0.05 M (pH 7.2) [28,53]. The nitrosylation test for catechols was performed by sequentially treating sections of fresh healthy needles with 1 volume of 10% sodium nitrite, 1 volume of 20% urea, 1 volume of 10% acetic acid, and 2 volumes of 2 M sodium hydroxide. In the presence of catechols, this reagent forms a red color which later turns brown after 1 h [54]. Sections of necrotic needles and healthy FAA fixed needles were treated with a 20% *w/v* solution of vanillin in ethanol for 3 min followed by concentrated HCl to test for the presence of condensed tannins, which produce a red coloration [55].

## 5. Conclusions

Autofluorescence was capable of detecting/distinguishing lignin, suberin, ferulate, flavonoids, extractives, and chlorophyll in fresh pine needle cross-sections using confocal microscopy and fluorescence spectroscopy. The occurrence of ferulate in phloem cell walls was variable, possibly indicating the stress state of the needle, whereas cutin fluorescence varied between the abaxial and adaxial surfaces of the needle. Extractives showed relatively weak fluorescence, but several different components could be detected. Chlorotic needles showed similar autofluorescence to healthy needles apart from the partial or complete loss of chlorophyll fluorescence. In necrotic needles, mesophyll extractives underwent a change in emission from weak blue fluorophores excited by UV to strong green fluorophores excited by blue light. This change was associated with the browning of needles. A similar change in extractive fluorescence occurred as a result of FAA fixation. Autofluorescence, therefore, provides a method for acquiring a single image that can be used to screen for multiple components of radiata pine needles in order to understand pathological changes and to potentially allow screening for resistance and disease progression.

## Figures and Tables

**Table 1:** Excitation and emission wavelengths for autofluorescent compounds in fresh pine needles.

Fluorophore	Excitation	Emission	λmax
Extractive (mesophyll)	355 nm	Blue	455 nm
Suberin	355 nm	Blue	465 nm
Lignin	355/488 nm	Blue/Green	455/535 nm
Ferulate	355 nm	Green (pH9)	480 nm
Flavonoid	488 nm	Green	535 nm
Chlorophyll	355/488/561/633 nm	Red	685/730

## Supplementary Materials

The following are available online at http://www.mdpi.com/2223-7747/7/1/10/s1, Figure S1: Spectra of extractives compared to pine bark tannin.
