# Supplementary material for: Imaging and Spectroscopy of Natural Fluorophores in Pine Needles
Source: Plants (Basel). 2018 Feb 2;7(1):10. doi: 10.3390/plants7010010 (PMC5874599; doi:10.3390/plants7010010)
Supplement: Supplementary file 1 [file plants-07-00010-s001.pdf]

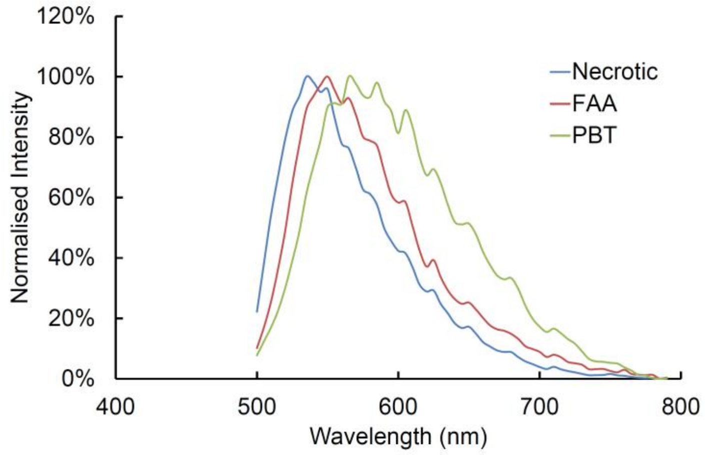

Figure S1. A comparison of fluorescence spectra from necrotic mesophyll, FAA fixed mesophyll extractives and pine bark tannin (PBT) using 488 nm excitation. The spectrum for FAA fixed mesophyll has been spectrally unmixed to remove the fluorescence from chlorophyll contamination. The spectrum for necrotic mesophyll is significantly different from that for FAA fixed mesophyll, $\chi^2 = 226.7$ $***$, and for pine bark tannin, $\chi^2 = 1944.9$ $***$.
